# Statistical Generalized Derivative Applied to the Profile Likelihood Estimation in a Mixture of Semiparametric Models

**DOI:** 10.3390/e22030278

**Published:** 2020-02-28

**Authors:** Yuichi Hirose, Ivy Liu

**Affiliations:** School of Mathematics and Statistics, Victoria University of Wellington, P.O. Box 600, 6140 Wellington, New Zealand; ivy.liu@vuw.ac.nz

**Keywords:** efficiency, efficient information bound, efficient score, implicitly defined function, profile likelihood, semi-parametric model, joint model, EM algorithm

## Abstract

There is a difficulty in finding an estimate of the standard error (SE) of the profile likelihood estimator in the joint model of longitudinal and survival data. The difficulty is on the differentiation of an implicit function that appear in the profile likelihood estimation. We solve the difficulty by introducing the “statistical generalized derivative”. The derivative is used to show the asymptotic normality of the estimator with the SE expressed in terms of the profile likelihood score function.

## 1. Introduction

This paper proposes a method to show asymptotic normality of the maximum profile likelihood estimator (profile likelihood MLE) in a mixture of semiparametric models with the EM-algorithm. We derive an expression for the standard error (SE) of the estimator using the profile likelihood score function. As an example, we consider a joint model of ordinal responses and the proportional hazards model with finite mixture. Through this example, we demonstrate solving the theoretical challenge in a joint model of survival and longitudinal data stated by [1]: “No distributional or asymptotic theory is available to date, and even the standard errors (SEs), defined as the standard deviations of the parametric estimators, are difficult to obtain.” The difficulty of the problem is to deal with an implicit function which is difficult to differentiate. In the profile likelihood approach, we profile out the baseline hazard function by plugging in an estimate of the hazard function to the likelihood function. This estimator of the hazard function is an implicit function in our problem (see also [2], p67).

Here, we review some of the related works. For a more complete review of the joint models please see [2] and [3]. The paper [4] proposed a profile likelihood approach to a joint model with unobserved random effects. The EM-algorithm was applied for an estimation of parameters in the model. This approach became one of the standard models and has been adopted by many (for example, [5,6,7]). In a series of studies, ([8,9,10,11]), also adopted a profile likelihood approach in [4] and showed the asymptotic normality of the profile likelihood MLE. As a result, they obtained the SE of the estimator. Another well known approach was proposed by [12]. They used "an approximate least favorable submodel" to show the asymptotic normality of the profile likelihood MLE. The SE of the profile likelihood estimator was obtained from the asymptotic normality.

All of the existing work mentioned here avoided dealing with the implicit function in the profile likelihood. For example, the works of [8,9,10,11] used the equality between the maximum likelihood estimator (MLE) and the maximum profile likelihood estimator (profile likelihood MLE). They have shown the asymptotic normality of the MLE in the joint models and the result is used to show the asymptotic normality of the profile likelihood MLE. This indirect proof avoided the differentiation of the implicit function in the profile likelihood. In the case of [12], "an approximate least favorable submodel" was used to approximate the profile likelihood function. Then, this approximation was used to prove the result without differentiating the profile likelihood function directly.

In summary, the existing methods showed the asymptotic normality of the profile likelihood MLE using methods to avoid differentiating a profile likelihood function. They have shown that the variance of the profile likelihood MLE is the inverse of an efficient information matrix. However, they have not shown the efficient information matrix in terms of the score function in the profile likelihood.

In this paper, we introduce "the statistical generalized derivative" to deal with the differentiation of implicit function in the profile likelihood under consideration. Our approach enabled us to expand the profile likelihood function to show the asymptotic normality of the profile likelihood MLE with the efficient information matrix expressed as a variance of the profile likelihood score function.

The results of this paper give us an analytical understanding of profile likelihood estimation and a method of computing the SE of the profile likelihood MLE in terms of the profile likelihood score function.

## 2. Mixture of Semiparametric Models and Generalized Statistical Derivative

### 2.1. Introduction of Mixture Model and Notations

We consider a mixture of semiparametric models whose density is of the form

(1) $$\begin{array}{c} p\left(x;\theta ,\eta ,\pi \right)=\sum_{r=1}^{R}\pi_{r}p_{r}\left(x;\theta_{r},\eta_{r}\right), \end{array}$$

where for each r=1,…,R, $p_{r}\left(x;\theta_{r},\eta_{r}\right)$ is a semiparametric model with a finite dimensional parameter $\theta_{r}\in \Theta_{r}\subset R^{m_{r}}$ and an infinite dimensional parameter $\eta_{r}\in H_{r}$ where $H_{r}$ is a subset of Banach space $B_{r}$, and $\pi_{1},\ldots ,\pi_{R}$ are mixture probabilities. We assume that $\pi_{r}>0$ for each *r* and $\sum_{r=1}^{R}\pi_{r}=1$. We denote $\theta =(\theta_{1},\ldots ,\theta_{R})$, $\eta =(\eta_{1},\ldots ,\eta_{R})$ and $\pi =(\pi_{1},\ldots ,\pi_{R})$. Once we observe iid data $X_{1},\ldots ,X_{n}$ from the mixture model, the joint probability function of the data $X=(X_{1},\ldots ,X_{n})$ is given by

(2) $$\begin{array}{c} p\left(X;\theta ,\eta ,\pi \right)=\prod_{i=1}^{n}\sum_{r=1}^{R}\pi_{r}p_{r}\left(X_{i};\theta_{r},\eta_{r}\right). \end{array}$$

We consider θ is the parameters of interest, and η and π are nuisance parameters.

To discuss the EM-algorithm, we further introduce notations (we use notations from [13]). Let $Z_{i}=\left(Z_{i1},\ldots ,Z_{iR}\right)$ be a group indicator variable for the subject *i*: for each *r*, $Z_{ir}=0$ or =1 with $P\left(Z_{ir}=1\right)=\pi_{r}$, and $\sum_{r=1}^{R}Z_{ir}=1$. Let $Z=(Z_{1},\ldots ,Z_{n})$. The joint probability function of the complete data (X,Z) is

(3) $$\begin{array}{c} p\left(X,Z;\theta ,\eta ,\pi \right)=\prod_{i=1}^{n}\prod_{r=1}^{R}{\left[\pi_{r}p_{r}\left(X_{i};\theta_{r},\eta_{r}\right)\right]}^{Z_{ir}}. \end{array}$$

Since $Z_{ir}$ are unobserved, it is common to replace with its expected value. The expected complete data log-likelihood under p(Z|X;θ,η,π) is

(4) $$\begin{array}{c} \sum_{i=1}^{n}\sum_{r=1}^{R}E\left(Z_{ir}\right)\left[\log\pi_{r}+\log p_{r}\left(X_{i};\theta_{r},\eta_{r}\right)\right]=\sum_{i=1}^{n}\sum_{r=1}^{R}\gamma \left(Z_{ir}\right)\left[\log\pi_{r}+\log p_{r}\left(X_{i};\theta_{r},\eta_{r}\right)\right]. \end{array}$$

where

(5) $$\begin{array}{c} \gamma \left(Z_{ir}\right)=\frac{\pi_{r}p_{r}\left(X_{i};\theta_{r},\eta_{r}\right)}{\sum_{j=1}^{R}\pi_{j}p_{j}\left(X_{i};\theta_{j},\eta_{j}\right)},\;\;r=1,\ldots ,R. \end{array}$$

### 2.2. Introducing Profile Likelihood, the Efficient Score Function and the Efficient Information Matrix

**The efficient score function and information matrix in the mixture model:** The score function for θ and score operator for η in the mixture model given in (1) are, respectively,

(6) $$\begin{array}{ccc} \dot{\ell }\left(x;\theta ,\eta \right) & = & \frac{\partial }{\partial \theta }\log\left(\sum_{r=1}^{R}\pi_{r}p_{r}\left(x;\theta_{r},\eta_{r}\right)\right)=\sum_{r=1}^{R}\gamma \left(z_{r}\right)\frac{\partial }{\partial \theta }\log p_{r}\left(x;\theta_{r},\eta_{r}\right), \end{array}$$

and

(7) $$\begin{array}{ccc} B(x;\theta ,\eta ) & = & d_{\eta }\log\left(\sum_{r=1}^{R}\pi_{r}p_{r}\left(x;\theta_{r},\eta_{r}\right)\right)=\sum_{r=1}^{R}\gamma \left(z_{r}\right)d_{\eta }\log p_{r}\left(x;\theta_{r},\eta_{r}\right) \end{array}$$

where $\gamma_{r}\left(z_{r}\right)$ is given in (5) with $Z_{ir}$ replaced with $z_{r}$. The notation $d_{\eta }$ is the Hadamard derivative operator with respect to the parameter η.

Let $\theta_{0},\eta_{0}$ be the true values of θ,η and denote ${\dot{\ell }}_{0}\left(x\right)=\dot{\ell }\left(x;\theta_{0},\eta_{0}\right)$ and $B_{0}\left(x\right)=B\left(x;\theta_{0},\eta_{0}\right)$. Then, it follows from the standard theory ([14], page 374) that the efficient score function ${\tilde{\ell }}_{0}$ and the efficient information matrix ${\tilde{I}}_{0}$ in the semiparametric mixture model are given by

(8) $$\begin{array}{c} {\tilde{\ell }}_{0}\left(x\right)=\left(I-B_{0}{\left(B_{0}^{*}B_{0}\right)}^{-1}B_{0}^{*}\right){\dot{\ell }}_{0}\left(x\right), \end{array}$$

and

(9) $$\begin{array}{c} {\tilde{I}}_{0}=E\left[{\tilde{\ell }}_{0}{\tilde{\ell }}_{0}^{T}\right]. \end{array}$$

Note: Equations (6) and (7) show that the score functions in the semiparametric mixture model (1) coincide with the ones for the expected complete data likelihood (4).

**Introduction of the profile likelihood and its score functions:** In the estimation of (θ,η), we use the profile likelihood approach. Let *F* be a cdf function which belongs to a set containing the empirical cdf $F_{n}$ and the true cdf $F_{0}$. In the profile likelihood approach, we obtain a function $\left(\theta ,F\right)\to {\hat{\eta }}_{\theta ,F}=\left({\hat{\eta }}_{1,\theta ,F},\ldots ,{\hat{\eta }}_{R,\theta ,F}\right)$ whose values are in the space of the parameter $\eta =(\eta_{1},\ldots ,\eta_{R})$. Then the profile likelihood function is defined by

(10) $$\begin{array}{c} p\left(X;\theta ,F,\pi \right)=\prod_{i=1}^{n}\sum_{r=1}^{R}\pi_{r}p_{r}\left(X_{i};\theta_{r},{\hat{\eta }}_{r,\theta ,F}\right). \end{array}$$

We also define the score functions for the profile likelihood in the model

(11) $$\begin{array}{c} \phi \left(x;\theta ,F\right)=\frac{\partial }{\partial \theta }\log\left(\sum_{r=1}^{R}\pi_{r}p_{r}\left(x;\theta_{r},{\hat{\eta }}_{r,\theta ,F}\right)\right)=\sum_{r=1}^{R}\gamma_{r}\left(z_{r}\right)\frac{\partial }{\partial \theta }\log p_{r}\left(x;\theta_{r},{\hat{\eta }}_{r,\theta ,F}\right) \end{array}$$

and

(12) $$\begin{array}{c} \psi \left(x;\theta ,F\right)=d_{F}\log\left(\sum_{r=1}^{R}\pi_{r}p_{r}\left(x;\theta_{r},{\hat{\eta }}_{r,\theta ,F}\right)\right)=\sum_{r=1}^{R}\gamma_{r}\left(z_{r}\right)d_{F}\log p_{r}\left(x;\theta_{r},{\hat{\eta }}_{r,\theta ,F}\right), \end{array}$$

where $\gamma_{r}\left(z_{r}\right)$ is given in (5) with $Z_{ir}$ replaced with $z_{r}$ and $\eta_{r}$ with ${\hat{\eta }}_{r,\theta ,F}$.

### 2.3. The EM-Algorithm to Obtain the Profile Likelihood MLE

Here, we describe the EM-algorithm applied to the profile likelihood function to obtain the profile likelihood MLE $\hat{\theta }$ of θ:

**The** **E-step:** In the E-step, we use the current parameter estimates $\hat{\theta }$ of θ to find the expected values of $Z_{ir}$:

(13) $$\begin{array}{c} \gamma \left(Z_{ir}\right)=\frac{\pi_{r}p_{r}\left(X_{i};{\hat{\theta }}_{r},{\hat{\eta }}_{r,\hat{\theta },F_{n}}\right)}{\sum_{j=1}^{R}\pi_{j}p_{j}\left(X_{i};{\hat{\theta }}_{j},{\hat{\eta }}_{j,\hat{\theta },F_{n}}\right)},\;\;r=1,\ldots ,R. \end{array}$$

**The** **M-step:**

**** In the M-step,

1. Calculate the estimates of $\pi_{r}$
  $$\begin{array}{ccc} \hat{\pi_{r}} & = & \frac{\sum_{i=1}^{n}\gamma \left(Z_{ir}\right)}{n}. \end{array}$$
2. Keeping $\gamma (Z_{ir})$ as a constant, we maximize the expected complete data log-likelihood
  (14) $$\begin{array}{c} \sum_{i=1}^{n}\sum_{r=1}^{R}\gamma \left(Z_{ir}\right)\log p_{r}\left(X_{i};\theta_{r},{\hat{\eta }}_{r,\theta ,F_{n}}\right). \end{array}$$
  with respect to θ to obtain new estimates $\hat{\theta }$.

The estimated parameters from the M-step are returned into the E-step until the value of $\hat{\theta }$ converges. The resulting estimator is the profile likelihood MLE.

### 2.4. Generalized Statistical Derivative and Asymptotic Normality of the Profile Likelihood MLE

In this section, we show the main result. We show the asymptotic normality of the profile likelihood MLE in Theorem 2. From the asymptotic normality, we can get an estimate of the SE of the estimator. Toward this goal, we introduce the statistical generalized derivative in Theorem 1.

**Assumptions:** We list assumptions used for Theorem 1 and Theorem 2 given below.

Let us denote $\theta_{0}$, $\eta_{0}$ and $F_{0}$ are the true values of the parameters θ, η and cdf *F*.

On the set of cdf functions F, we use the sup-norm, i.e. for $F,F_{0}\in F$,

$$∥F-F_{0}∥=\underset{x}{\sup}\left|F\left(x\right)-F_{0}\left(x\right)\right|.$$

For ρ>0, let

$$C_{\rho }=\{F\in F:∥F-F_{0}∥<\rho \}.$$

We denote the density function for the profile likelihood in the mixture model by

(15) $$p\left(x;\theta ,F\right)=\sum_{r=1}^{R}\pi_{r}p_{r}\left(x;\theta_{r},{\hat{\eta }}_{r,\theta ,F}\right).$$

We assume that:

(R1) The density function p(x;θ,F) is bounded away from 0, i.e., there is a constant c>0 such that for each *x* and (θ,F)∈Θ×F, p(x;θ,F)>c>0. More over, the density function p(x;θ,F) is continuously differentiable with respect to θ and Hadamard differentiable with respect to *F* for all *x*. We denote derivatives by $\phi \left(x;\theta ,F\right)=\frac{\partial }{\partial \theta }\log p\left(x;\theta ,F\right)$ and $\psi \left(x;\theta ,F\right)=d_{F}\log p\left(x;\theta ,F\right)$ and they are given in (11) and (12).
(R2) We assume ${\hat{\eta }}_{\theta ,F}$ satisfies ${\hat{\eta }}_{\theta_{0},F_{0}}=\eta_{0}$ and the function
  $${\tilde{\ell }}_{0}\left(x\right):=\phi \left(x;\theta_{0},F_{0}\right)$$
  is the efficient score function. Further, we assume $n^{1/2}\left(F_{n}-F_{0}\right)=O_{P}\left(1\right)$ and $\left({\hat{\eta }}_{{\hat{\theta }}_{n},F_{n}}-\eta_{0}\right)=o_{P}\left(1\right)$ if ${\hat{\theta }}_{n}$ is a consistent estimator of $\theta_{0}$.
(R3) The efficient information matrix ${\tilde{I}}_{0}=E\left[{\tilde{\ell }}_{0}{\tilde{\ell }}_{0}^{T}\right]=E\left[\phi \phi^{T}\left(X;\theta_{0},F_{0}\right)\right]$ is inevitable.
(R4) The score function ϕ(x;θ,F) defined in (11) takes the form
  (16) $$\phi \left(x;\theta ,F\right)=\tilde{\phi }\left(x;\theta ,F,{\hat{\eta }}_{\theta ,F}\right),$$
  where, by assumption (R2), the efficient score function is given by
  $${\tilde{\ell }}_{0}\left(x\right)=\phi \left(x;\theta_{0},F_{0}\right)=\tilde{\phi }\left(x;\theta_{0},F_{0},\eta_{0}\right).$$
  We assume that there exists a ρ>0 and neighborhoods Θ and *H* of $\theta_{0}$ and $\eta_{0}$, respectively, such that $C_{\rho }$ and *H* are Donsker and the class of functions $\left\{\tilde{\phi }\left(x;\theta ,F,\eta \right):\;\left(\theta ,F,\eta \right)\in \Theta \times C_{\rho }\times H\right\}$ has a square integrable envelope function and it is Lipschitz in the parameters (θ,F,η):
  (17) $$\begin{array}{c} ∥\tilde{\phi }\left(x;\theta^{\prime },F^{\prime },\eta^{\prime }\right)-\tilde{\phi }\left(x;\theta ,F,\eta \right)∥\leq M^{\prime }\left(x\right)(∥\theta^{\prime }-\theta ∥+∥F^{\prime }-F∥+∥\eta^{\prime }-\eta ∥) \end{array}$$
  where $M^{\prime }\left(x\right)$ is a $P_{0}$-square integrable function.

Note: Since the assumption (R4) is unusual condition, it requires an explanation. In our profile likelihood problem the estimate ${\hat{\eta }}_{\theta ,F}$ of the parameter η is an implicit function. Therefore, the map $\left(\theta ,F\right)\to {\hat{\eta }}_{\theta ,F}$ is hard to differentiate. It follows that the profile likelihood score function ϕ(x;θ,F) defined in (11) is hard to differentiate. However it is often the case that the function ϕ(x;θ,F) takes the form in (16). In the examples, the map $\left(\theta ,\eta ,F\right)\to \tilde{\phi }\left(x;\theta ,F,\eta \right)$ is in a closed form and therefore it is easy to differentiate. This differentiability became handy when we need to expand the function ϕ(x;θ,F) through the differentiability of $\tilde{\phi }\left(x;\theta ,F,\eta \right)$. We use the assumption (R4) in the proof of Equation (20) in Theorem 1. In the example in Section 3, the function in (44) corresponds to ${\hat{\eta }}_{\theta ,F}$ and the function in (51) corresponds to $\tilde{\phi }\left(x;\theta ,F,\eta \right)$.

Note: To calculate the second derivative of the score function ϕ(x;θ,F) given in (11), we use the idea similar to the derivative of generalized functions ([15]). Let $\varphi \to \left(f,\varphi \right)=\int_{-\infty }^{\infty }f\left(x\right)\varphi \left(x\right)dx$ be a generalized function, where φ vanishes outside of some interval. Then if *f* and φ are differentiable with derivative $f^{\prime }$ and $\varphi^{\prime }$, then by integration by parts,

$$\left(f^{\prime },\varphi \right)=\int_{-\infty }^{\infty }f^{\prime }\left(x\right)\varphi \left(x\right)dx=-\int_{-\infty }^{\infty }f\left(x\right)\varphi^{\prime }\left(x\right)dx=-\left(f,\varphi^{\prime }\right).$$

We define the derivative $\left(f^{\prime },\varphi \right)$ of the generalized function φ→(f,φ) by $-(f,\varphi^{\prime })$. This definition is valid even if *f* is not differentiable, provided φ is differentiable.

A similar idea can be applied in our problem. Suppose the density for the profile likelihood p(x;θ,F) given in (10) is twice differentiable with respect to θ, then by differentiating

$$\begin{array}{c} \int \left\{\frac{\partial }{\partial \theta }\log p\left(x;\theta ,F\right)\right\}p\left(x;\theta ,F\right)dx=0, \end{array}$$

with respect to θ at $\left(\theta ,F\right)=\left(\theta_{0},F_{0}\right)$, we get equivalent expressions for the efficient information matrix in terms of the score function $\phi (x;\theta_{0},F_{0})$:

(18) $$\begin{array}{c} {\tilde{I}}_{0}=E\left[\phi \phi^{T}\left(X;\theta_{0},F_{0}\right)\right]=-E\left[\frac{\partial }{\partial \theta^{T}}\phi \left(X;\theta_{0},F_{0}\right)\right]. \end{array}$$

From this equation we are motivated to define the expected derivative of the score function $-E\left[\frac{\partial }{\partial \theta^{T}}\phi \left(X;\theta_{0},F_{0}\right)\right]$ by $E[\phi \phi^{T}\left(X;\theta_{0},F_{0}\right)]$. In the following theorem, we show that the definition is valid even when the derivative of the score function $\frac{\partial }{\partial \theta^{T}}\phi \left(x;\theta ,F\right)$ does not exist.

**Theorem** **1.** *(Statistical generalized derivative) Let $p\left(x;\theta ,F\right)=\sum_{r=1}^{R}\pi_{r}p_{r}\left(x;\theta_{r},{\hat{\eta }}_{r,\theta ,F}\right)$, $\phi \left(x;\theta ,F\right)=\frac{\partial }{\partial \theta }\log p\left(x;\theta ,F\right)$, and $\psi \left(x;\theta ,F\right)=d_{F}\log p\left(x;\theta ,F\right)$ as defined in (15), (11) and (12), respectively.*

*Suppose (R1) and (R4), then, for $\theta_{t}\to \theta_{0}$ and $F_{t}\to F_{0}$ as t→0, we have that*

(19) $$\begin{array}{cc} E & \left[t^{-1}\left\{\phi \left(X;\theta_{t},F_{0}\right)-\phi \left(X;\theta_{0},F_{0}\right)\right\}\right]\\ & =-E\left[\phi \left(X;\theta_{0},F_{0}\right)\phi^{T}\left(X;\theta_{0},F_{0}\right)\right]\left\{t^{-1}\left(\theta_{t}-\theta_{0}\right)\right\}+o\left(1\right), \end{array}$$

*and*

(20) $$\begin{array}{cc} E & \left[t^{-1}\left\{\phi \left(X;\theta_{t},F_{t}\right)-\phi \left(X;\theta_{t},F_{0}\right)\right\}\right]\\ & =-E\left[\phi \left(X;\theta_{0},F_{0}\right)\psi \left(X;\theta_{0},F_{0}\right)\right]\left\{t^{-1}\left(F_{t}-F_{0}\right)\right\}\\ + & O\{t^{-1}(∥\theta_{t}-\theta_{0}∥+∥F_{t}-F_{0}∥)(∥F_{t}-F_{0}∥+∥{\hat{\eta }}_{\theta_{t},F_{t}}-{\hat{\eta }}_{\theta_{t},F_{0}}∥)\}\\ + & o(1+∥\theta_{t}-\theta_{0}∥+∥F_{t}-F_{0}∥+∥{\hat{\eta }}_{\theta_{t},F_{t}}-\eta_{0}∥). \end{array}$$

Note: Note that even when the derivative $\frac{\partial }{\partial \theta }\phi \left(x;\theta ,F\right)$ does not exist the Equation (19) shows that the derivative of the map $\theta \to E\left[\phi (x;\theta ,F)\right]$ exists. We may call the derivative the statistical generalized derivative. A similar comment for (20) holds.

**Proof.**  In (R1) we assumed the density function p(x;θ,F) is bounded away from 0 and it is differentiable with respect to θ and *F*. It follows that

(21) $$\begin{array}{c} \frac{t^{-1}\{p\left(x;\theta_{t},F_{0}\right)-p\left(x;\theta_{0},F_{0}\right)}{p(x;\theta_{0},F_{0})}=\phi \left(x;\theta_{0},F_{0}\right)\left\{t^{-1}\left(\theta_{t}-\theta_{0}\right)\right\}+o\left(1\right), \end{array}$$

(22) $$\begin{array}{c} \frac{t^{-1}\{p\left(x;\theta_{t},F_{t}\right)-p\left(x;\theta_{t},F_{0}\right)}{p(x;\theta_{t},F_{0})}=\psi \left(x;\theta_{t},F_{0}\right)\left\{t^{-1}\left(F_{t}-F_{0}\right)\right\}+o\left(1\right), \end{array}$$

and

(23) $$\begin{array}{c} \frac{t^{-1}\left\{p\left(x;\theta_{t},F_{t}\right)-p\left(x;\theta_{0},F_{0}\right)\right\}}{p(x;\theta_{0},F_{0})}=\phi \left(x;\theta_{0},F_{0}\right)t^{-1}\left(\theta_{t}-\theta_{0}\right)+\psi \left(x;\theta_{0},F_{0}\right)t^{-1}\left(F_{t}-F_{0}\right)+o\left(1\right). \end{array}$$

We prove the first Equation (19). For each *t*, the equality

$$\begin{array}{ccc} 0 & = & t^{-1}\left\{\int \phi \left(x;\theta_{t},F_{0}\right)p\left(x;\theta_{t},F_{0}\right)dx-\int \phi \left(x;\theta_{0},F_{0}\right)p\left(x;\theta_{0},F_{0}\right)dx\right\}\\ & = & \int t^{-1}\left\{\phi \left(x;\theta_{t},F_{0}\right)-\phi \left(x;\theta_{0},F_{0}\right)\right\}p\left(x;\theta_{0},F_{0}\right)dx\\ & & +\int \phi \left(x;\theta_{t},F_{0}\right)t^{-1}\left\{p\left(x;\theta_{t},F_{0}\right)-p\left(x;\theta_{0},F_{0}\right)\right\}dx \end{array}$$

holds. It follows that, for each *t*, we have that

(24) $$\begin{array}{cc} \int t^{-1} & \left\{\phi \left(x;\theta_{t},F_{0}\right)-\phi \left(x;\theta_{0},F_{0}\right)\right\}p\left(x;\theta_{0},F_{0}\right)dx\\ & =-\int \phi \left(x;\theta_{t},F_{0}\right)t^{-1}\left\{p\left(x;\theta_{t},F_{0}\right)-p\left(x;\theta_{0},F_{0}\right)\right\}dx. \end{array}$$

By the dominated convergence theorem with (21), the right hand side of (24) is, as t→0,

$$\begin{array}{cc} & -\int \phi \left(x;\theta_{t},F_{0}\right)t^{-1}\left\{p\left(x;\theta_{t},F_{0}\right)-p\left(x;\theta_{0},F_{0}\right)\right\}dx\\ = & -\int \phi \left(x;\theta_{t},F_{0}\right)\frac{t^{-1}\left\{p\left(x;\theta_{t},F_{0}\right)-p\left(x;\theta_{0},F_{0}\right)\right\}}{p(x;\theta_{0},F_{0})}p\left(x;\theta_{0},F_{0}\right)dx\\ = & -\int \phi \left(x;\theta_{0},F_{0}\right)\phi^{T}\left(x;\theta_{0},F_{0}\right)p\left(x;\theta_{0},F_{0}\right)dx\left\{t^{-1}\left(\theta_{t}-\theta_{0}\right)\right\}+o\left(1\right). \end{array}$$

It follows that, we have (19):

$$\begin{array}{cc} \int t^{-1} & \left\{\phi \left(x;\theta_{t},F_{0}\right)-\phi \left(x;\theta_{0},F_{0}\right)\right\}p\left(x;\theta_{0},F_{0}\right)dx\\ & =-\int \phi \left(x;\theta_{0},F_{0}\right)\phi^{T}\left(x;\theta_{0},F_{0}\right)p\left(x;\theta_{0},F_{0}\right)dx\left\{t^{-1}\left(\theta_{t}-\theta_{0}\right)\right\}+o\left(1\right). \end{array}$$

Now we prove the second Equation (20). Similar to the beginning of the proof of (19), for each *t*, the following equation holds:

(25) $$\begin{array}{cc} \int & t^{-1}\left\{\phi \left(x;\theta_{t},F_{t}\right)-\phi \left(x;\theta_{t},F_{0}\right)\right\}p\left(x;\theta_{t},F_{t}\right)dx\\ & =-\int \phi \left(x;\theta_{t},F_{0}\right)t^{-1}\left\{p\left(x;\theta_{t},F_{t}\right)-p\left(x;\theta_{t},F_{0}\right)\right\}dx. \end{array}$$

Using (23) with the dominated convergence theorem, the left hand side of (25) is, as t→0,

(26) $$\begin{array}{cc} & ∥\int t^{-1}\left\{\phi \left(x;\theta_{t},F_{t}\right)-\phi \left(x;\theta_{t},F_{0}\right)\right\}p\left(x;\theta_{t},F_{t}\right)dx-\int t^{-1}\left\{\phi \left(x;\theta_{t},F_{t}\right)-\phi \left(x;\theta_{t},F_{0}\right)\right\}p\left(x;\theta_{0},F_{0}\right)dx∥\\ = & ∥\int \left\{\phi \left(x;\theta_{t},F_{t}\right)-\phi \left(x;\theta_{t},F_{0}\right)\right\}\frac{t^{-1}\left\{p\left(x;\theta_{t},F_{t}\right)-p\left(x;\theta_{0},F_{0}\right)\right\}}{p(x;\theta_{0},F_{0})}p\left(x;\theta_{0},F_{0}\right)dx∥\\ \leq & \left\{∥\int M^{\prime }\left(x\right)\left\{\phi \left(x;\theta_{0},F_{0}\right)t^{-1}\left(\theta_{t}-\theta_{0}\right)+\psi \left(x;\theta_{0},F_{0}\right)t^{-1}\left(F_{t}-F_{0}\right)\right\}p\left(x;\theta_{0},F_{0}\right)dx∥+o\left(1\right)\right\}\\ & \times (∥F_{t}-F_{0}∥+∥{\hat{\eta }}_{\theta_{t},F_{t}}-{\hat{\eta }}_{\theta_{t},F_{0}}∥)\\ = & O\{t^{-1}(∥\theta_{t}-\theta_{0}∥+∥F_{t}-F_{0}∥)(∥F_{t}-F_{0}∥+∥{\hat{\eta }}_{\theta_{t},F_{t}}-{\hat{\eta }}_{\theta_{t},F_{0}}∥)\}+o(∥F_{t}-F_{0}∥+∥{\hat{\eta }}_{\theta_{t},F_{t}}-{\hat{\eta }}_{\theta_{t},F_{0}}∥), \end{array}$$

where we used (17): with a $P_{0}$-square integrable function $M^{\prime }\left(x\right)$,

$$\begin{array}{ccc} ∥\phi \left(x;\theta_{t},F_{t}\right)-\phi \left(x;\theta_{t},F_{0}\right)∥ & = & ∥\tilde{\phi }\left(x;\theta_{t},F_{t},{\hat{\eta }}_{\theta_{t},F_{t}}\right)-\tilde{\phi }\left(x;\theta_{t},F_{0},{\hat{\eta }}_{\theta_{t},F_{0}}\right)∥\\ & \leq & M^{\prime }\left(x\right)(∥F_{t}-F_{0}∥+∥{\hat{\eta }}_{\theta_{t},F_{t}}-{\hat{\eta }}_{\theta_{t},F_{0}}∥). \end{array}$$

By the dominated convergence theorem with (22), it follows that the integral in the right hand side of the Equation (25) is

(27) $$\begin{array}{cc} & \int \phi \left(x;\theta_{t},F_{0}\right)t^{-1}\left\{p\left(x;\theta_{t},F_{t}\right)-p\left(x;\theta_{t},F_{0}\right)\right\}dx\\ = & \int \phi \left(x;\theta_{0},F_{0}\right)\psi \left(x;\theta_{0},F_{0}\right)t^{-1}\left(F_{t}-F_{0}\right)p\left(x;\theta_{0},F_{0}\right)dx\\ & +O\{t^{-1}∥F_{t}-F_{0}∥(∥\theta_{t}-\theta_{0}∥+∥{\hat{\eta }}_{\theta_{t},F_{0}}-\eta_{0}∥)\}+o(1+(∥\theta_{t}-\theta_{0}∥+∥{\hat{\eta }}_{\theta_{t},F_{0}}-\eta_{0}∥)), \end{array}$$

here again we used (17): there is a $P_{0}$-square integrable function $M^{\prime }\left(x\right)$ such that

$$\begin{array}{ccc} ∥\phi \left(x;\theta_{t},F_{0}\right)-\phi \left(x;\theta_{0},F_{0}\right)∥ & = & ∥\tilde{\phi }\left(x;\theta_{t},F_{0},{\hat{\eta }}_{\theta_{t},F_{0}}\right)-\tilde{\phi }\left(x;\theta_{0},F_{0},\eta_{0}\right)∥\\ & \leq & M^{\prime }\left(x\right)(∥\theta_{t}-\theta_{0}∥+∥{\hat{\eta }}_{\theta_{t},F_{0}}-\eta_{0}∥). \end{array}$$

Since $O\left({\hat{\eta }}_{\theta_{t},F_{t}}-{\hat{\eta }}_{\theta_{t},F_{0}}\right)=O\left({\hat{\eta }}_{\theta_{t},F_{0}}-{\hat{\eta }}_{\theta_{t},F_{0}}\right)=O\left({\hat{\eta }}_{\theta_{t},F_{t}}-\eta_{0}\right)$, by combining (26) and (27), the equality (25) is equivalent to

$$\begin{array}{cc} & \int t^{-1}\left\{\phi \left(x;\theta_{t},F_{t}\right)-\phi \left(x;\theta_{t},F_{0}\right)\right\}p\left(x;\theta_{0},F_{0}\right)dx\\ = & -\int \phi \left(x;\theta_{0},F_{0}\right)\psi \left(x;\theta_{0},F_{0}\right)p\left(x;\theta_{0},F_{0}\right)dx\left\{t^{-1}\left(F_{t}-F_{0}\right)\right\}\\ & +O\{t^{-1}(∥\theta_{t}-\theta_{0}∥+∥F_{t}-F_{0}∥)(∥F_{t}-F_{0}∥+∥{\hat{\eta }}_{\theta_{t},F_{t}}-{\hat{\eta }}_{\theta_{t},F_{0}}∥)\}\\ & +o(1+∥\theta_{t}-\theta_{0}∥+∥F_{t}-F_{0}∥+∥{\hat{\eta }}_{\theta_{t},F_{t}}-\eta_{0}∥). \end{array}$$

The (20) follows from this.  □

Using the result in Theorem 1, we show the asymptotic normality of the profile likelihood MLE:

**Theorem** **2.** *(Asymptotic normality of the profile likelihood MLE) Suppose the set of assumptions (R1)−(R4) holds. If the profile likelihood MLE (described in Section 2.3) is consistent, then it is an asymptotically linear estimator for $\theta_{0}$:*

$$\sqrt{n}\left({\hat{\theta }}_{n}-\theta_{0}\right)=\frac{1}{\sqrt{n}}\sum_{i=1}^{n}{\tilde{I}}_{0}^{-1}{\tilde{\ell }}_{0}\left(X_{i}\right)+o_{P}\left(1\right).$$

*Hence we have that*

$$\sqrt{n}\left({\hat{\theta }}_{n}-\theta_{0}\right)\overset{d}{⟶}N\left(0,{\tilde{I}}_{0}^{-1}\right)\;\;\mathrm{as}n\to \infty .$$

**Proof.**  The profile likelihood MLE described in Section 2.3 is solution to the estimating equation

(28) $$\begin{array}{c} \sum_{i=1}^{n}\phi \left(X_{i};{\hat{\theta }}_{n},F_{n}\right)=0 \end{array}$$

where ϕ(x;θ,F) is the profile likelihood score function for θ defined in (11).

In (R4) we assumed $C_{\rho }$ and *H* are Donsker and the function $\tilde{\phi }\left(x;\theta ,F,\eta \right)$ is Lipschitz in the parameters (θ,F,η) with a $P_{0}$-square integrable function $M^{\prime }\left(x\right)$ given in (17). By Corollary 2.10.13 in [16], the class $\left\{\tilde{\phi }\left(x;\theta ,F,\eta \right):\;\left(\theta ,F,\eta \right)\in \Theta \times C_{\rho }\times H\right\}$ is Donsker. Moreover, we assumed ${\hat{\theta }}_{n}-\theta_{0}=o_{p}\left(1\right)$. In (R2), we assumed $n^{1/2}\left(F_{n}-F_{0}\right)=O_{p}\left(1\right)$ and ${\hat{\eta }}_{{\hat{\theta }}_{n},F_{n}}-\eta_{0}=o_{p}\left(1\right)$. By the dominated convergence theorem with (17), we have $E\left[{\left(\tilde{\phi }\left(X;{\hat{\theta }}_{n},F_{n},{\hat{\eta }}_{{\hat{\theta }}_{n},F_{n}}\right)-\tilde{\phi }\left(X;\theta_{0},F_{0},\eta_{0}\right)\right)}^{2}\right]=o_{p}\left(1\right)$.

By Lemma 19.24 in [14], it follows that

$$\begin{array}{c} \frac{1}{\sqrt{n}}\sum_{i=1}^{n}\left\{\tilde{\phi }\left(X_{i};{\hat{\theta }}_{n},F_{n},{\hat{\eta }}_{{\hat{\theta }}_{n},F_{n}}\right)-\tilde{\phi }\left(X_{i};\theta_{0},F_{0},\eta_{0}\right)\right\}=\sqrt{n}E\left\{\tilde{\phi }\left(X;{\hat{\theta }}_{n},F_{n},{\hat{\eta }}_{{\hat{\theta }}_{n},F_{n}}\right)-\tilde{\phi }\left(X;\theta_{0},F_{0},\eta_{0}\right)\right\}+o_{P}\left(1\right). \end{array}$$

Using (16), this is equivalent to

(29) $$\begin{array}{c} \frac{1}{\sqrt{n}}\sum_{i=1}^{n}\left\{\phi \left(X_{i};{\hat{\theta }}_{n},F_{n}\right)-\phi \left(X_{i};\theta_{0},F_{0}\right)\right\}=\sqrt{n}E\left\{\phi \left(X;{\hat{\theta }}_{n},F_{n}\right)-\phi \left(X;\theta_{0},F_{0}\right)\right\}+o_{P}\left(1\right). \end{array}$$

From (19) it follows that

(30) $$\begin{array}{ccc} \sqrt{n}E\left\{\phi \left(X;{\hat{\theta }}_{n},F_{0}\right)-\phi \left(X;\theta_{0},F_{0}\right)\right\} & = & -{\tilde{I}}_{0}\sqrt{n}\left({\hat{\theta }}_{n}-\theta_{0}\right)+o_{p}\left(1\right), \end{array}$$

where ${\tilde{I}}_{0}=E\left[{\tilde{\ell }}_{0}{\tilde{\ell }}_{0}^{T}\right]=E\left\{\phi \left(X;\theta_{0},F_{0}\right)\phi^{T}\left(X;\theta_{0},F_{0}\right)\right\}$.

Using (20),

(31) $$\begin{array}{cc} & \sqrt{n}E\left\{\phi \left(X;{\hat{\theta }}_{n},F_{n}\right)-\phi \left(X;{\hat{\theta }}_{n},F_{0}\right)\right\}\\ = & -E\left[\phi \left(X;\theta_{0},F_{0}\right)\psi \left(X;\theta_{0},F_{0}\right)\right]\left\{\sqrt{n}\left(F_{n}-F_{0}\right)\right\}\\ & +O\{\sqrt{n}(∥{\hat{\theta }}_{n}-\theta_{0}∥+∥F_{n}-F_{0}∥)(∥F_{n}-F_{0}∥+∥{\hat{\eta }}_{{\hat{\theta }}_{n},F_{n}}-\eta_{0}∥)\}\\ & +o(1+∥{\hat{\theta }}_{n}-\theta_{0}∥+∥F_{n}-F_{0}∥+∥{\hat{\eta }}_{{\hat{\theta }}_{n},F_{n}}-\eta_{0}∥)\\ = & o_{P}\left(1+\sqrt{n}\left({\hat{\theta }}_{n}-\theta_{0}\right)\right), \end{array}$$

where we used:

1. Since $\psi (x;\theta_{0},F_{0})$ is in the nuisance tangent space and $\phi (x;\theta_{0},F_{0})$ is the efficient score function, we have
  (32) $$\begin{array}{c} E[\phi \left(x;\theta_{0},F_{0}\right)\psi \left(x;\theta_{0},F_{0}\right)]=0. \end{array}$$
2. Using consistency of ${\hat{\theta }}_{n}$ with assumptions $n^{1/2}\left(F_{n}-F_{0}\right)=O_{P}\left(1\right)$ and $\left({\hat{\eta }}_{{\hat{\theta }}_{n},F_{n}}-\eta_{0}\right)=o_{P}\left(1\right)$ in (R2), it follows that
  $$\begin{array}{ccc} O\{\sqrt{n}(∥{\hat{\theta }}_{n}-\theta_{0}∥+∥F_{n}-F_{0}∥)(∥F_{n}-F_{0}∥+∥{\hat{\eta }}_{{\hat{\theta }}_{n},F_{n}}-\eta_{0}∥)\} & = & o_{P}\left(1+\sqrt{n}\left({\hat{\theta }}_{n}-\theta_{0}\right)\right)\\ \;\mathrm{and}\;o(1+∥{\hat{\theta }}_{n}-\theta_{0}∥+∥F_{n}-F_{0}∥+∥{\hat{\eta }}_{{\hat{\theta }}_{n},F_{n}}-\eta_{0}∥) & = & o_{P}\left(1\right). \end{array}$$

Using (30) and (31), the right hand side of (29) is

(33) $$\begin{array}{cc} & \sqrt{n}E\left\{\phi \left(X;{\hat{\theta }}_{n},F_{n}\right)-\phi \left(X;\theta_{0},F_{0}\right)\right\}\\ = & \sqrt{n}E\left\{\phi \left(X;{\hat{\theta }}_{n},F_{0}\right)-\phi \left(X;\theta_{0},F_{0}\right)\right\}+\sqrt{n}E\left\{\phi \left(X;{\hat{\theta }}_{n},F_{n}\right)-\phi \left(X;{\hat{\theta }}_{n},F_{0}\right)\right\}\\ = & -{\tilde{I}}_{0}\sqrt{n}\left({\hat{\theta }}_{n}-\theta_{0}\right)+o_{p}\left\{1+\sqrt{n}\left({\hat{\theta }}_{n}-\theta_{0}\right)\right\}. \end{array}$$

Finally, (29) together with (33) and $\frac{1}{\sqrt{n}}\sum_{i=1}^{n}\phi \left(X_{i};{\hat{\theta }}_{n},F_{n}\right)=0$ imply that

$$\begin{array}{c} \sqrt{n}\left({\hat{\theta }}_{n}-\theta_{0}\right)=\frac{1}{\sqrt{n}}\sum_{i=1}^{n}{\tilde{I}}_{0}^{-1}\phi \left(X_{i};\theta_{0},F_{0}\right)+o_{P}\left(1\right). \end{array}$$

 □

## 3. Joint Mixture Model of Survival and Longitudinal Ordered Data

In the paper [17], we treated “the joint model of ordinal responses and the proportional hazards with the finite mixture” with real data and simulated data examples. In that paper we had the method of calculating the SE of the profile likelihood MLE applied to the examples. However it was not justified with proofs. This is the motivation to write this paper to give the proofs. In this section, we use the theorem 1 and theorem 2 to show the asymptotic normality of the profile likelihood MLE to the model and prove the method of calculating SE in the paper [17]. Please see [17] for real data and simulated data examples.

**Ordinal** **Response** **Models:** Let $Y_{ijm}$ be the ordered categorical response from 1 (poor) to *L* (excellent) on item (or question) *j* for subject *i* at the $m^{th}$ protocol-specified time point, where i=1,2,…,n, j=1,2,…,J and m=1,2,…,M. In total, there are *J* items in the questionnaire related to patients quality of life, collected at times $t_{1},t_{2},\ldots ,t_{M}$. Given that subject *i* belongs to group *r*, an ordered stereotype model can be written as

$$\begin{array}{c} \log\left[\frac{P(Y_{ijm}=\ell \;|\;\theta_{r})}{P(Y_{ijm}=1\;|\;\theta_{r})}\right]=a_{\ell }+\phi_{\ell }\left(b_{j}+\theta_{r}\right),\;\;\;r=1,\ldots ,R, \end{array}$$

where $a_{\ell }$ is a response level intercept parameter with ℓ=2,…,L, $b_{j}$ is an item effect, and $\theta_{r}$ is associated with the discrete latent variable, with $a_{1}=0$, $b_{1}=0$, $\phi_{1}=0$ and $\theta_{1}=0$. The parameter $\theta_{r}$ can be referred to as a group effect of the quality of life for patients in group *r*. However, the group memberships are unknown. The $\left\{\phi_{\ell }\right\}$ parameters can be regarded as unknown scores for the outcome categories. Because $\phi_{\ell }\left(b_{j}+\theta_{r}\right)=\left(A\phi_{\ell }\left(\left(b_{j}+\theta_{r}\right)/A\right)\right)$ for any constant A≠0, for identifiability, we need to impose monotone scores on $\left\{\phi_{\ell }\right\}$ to treat $Y_{ijm}$ as ordinal. Therefore, the model has the constraint $0=\phi_{1}\leq \phi_{2}\leq \ldots \leq \phi_{L}=1$. The ordinal response part of likelihood function for the *i*th subject is

(34) $$\begin{array}{ccc} P(Y_{i}\;|\;\theta_{r},\alpha )\; & = & \;\prod_{m=1}^{M_{i}}\prod_{j=1}^{J}\prod_{\ell =1}^{L}{\left(\frac{\exp(a_{\ell }+\phi_{\ell }\left(b_{j}+\theta_{r}\right))}{1+\sum_{k=2}^{L}\exp\left(a_{k}+\phi_{k}\left(b_{j}+\theta_{r}\right)\right)}\right)}^{Y_{ijm\ell }} \end{array}$$

where α=(a,b,ϕ). Each follow-up time point may have a different number of observations because some patient responses are missing.

**The** **Cox** **Proportional** **Hazards** **Model:** We consider the Cox proportional hazards model for the survival part in the joint model. Let *X* be a time-independent covariate. The hazard function for the failure time $T_{i}$ of the $i^{th}$ subject is of the form

(35) $$\begin{array}{ccc} \lambda (t|X_{i},\theta_{r},\delta ) & = & \lambda_{0}\left(t\right)\exp\left(\theta_{r}\delta_{0}+X_{i}\delta_{1}\right) \end{array}$$

where $\lambda_{0}\left(t\right)$ is the baseline hazard function. The latent variable $\theta_{r}$ is linked with the ordinal response model and $\delta =(\delta_{0},\delta_{1})$ are coefficients.

For the estimation of the baseline hazard function $\lambda_{0}\left(t\right)$, we use the method of nonparametric maximum likelihood described in [18] (Section 4.3). Let $\lambda_{i}$ be the hazard at time $t_{i}$, where $t_{1}<t_{2}<\ldots <t_{n}$ are the ordered observed times. Assume that the hazard is zero between adjacent times so that the survival time is discrete. The corresponding cumulative hazard function $\Lambda_{0}\left(t_{i}\right)=\sum_{p\leq i}\lambda_{p}$ is a step function with jumps at the failure time $t_{i}$. Then the survival part likelihood function of subject *i* is

(36) $$\begin{array}{ccc} P\left(T_{i},d_{i}\;|\;\lambda ,\theta_{r},\delta \right) & = & \;{\left(\lambda_{i}\exp\left(\theta_{r}\delta_{0}+X_{i}\delta_{1}\right)\right)}^{d_{i}}\times \exp\left(-\sum_{p\leq i}\lambda_{p}\exp\left(\theta_{r}\delta_{0}+X_{i}\delta_{1}\right)\right), \end{array}$$

where the $d_{i}$ is an indicator of censorship for individual *i*: if we observe failure time, then $d_{i}=1$, otherwise $d_{i}=0$.

**The** **Full** **Likelihood** **Function:** The joint likelihood function is obtained by combining the probability function from ordinal response model (34), and the proportional hazards model (36), by assuming the two models are independent given latent discrete random variables.

Let $\pi_{r}$ be the unknown probability (r=1,…,R) that a subject lies in group *r*, and (Θ,λ)=((θ,α,δ),λ) be all the unknown parameters of the joint model. The mixture model likelihood function is

(37) $$\begin{array}{c} L\left(\Theta ,\lambda |Y,T,D\right)=\prod_{i=1}^{n}\left(\sum_{r=1}^{R}P\left(Y_{i}\;|\;\theta_{r},\alpha \right)P\left(T_{i},d_{i}\;|\;\lambda ,\theta_{r},\delta \right)\pi_{r}\right). \end{array}$$

Let $Z_{ir}$ be the group indicator, where $Z_{ir}$ = 1 if the $i^{th}$ individual was from the $r^{th}$ group and 0 otherwise. The complete data likelihood can be written as

(38) $$\begin{array}{c} L\left(\Theta ,\lambda |Y,T,d,Z\right)=\prod_{i=1}^{n}\prod_{r=1}^{R}{\left(\;P\left(Y_{i}\;|\;\theta_{r},\alpha \right)P\left(T_{i},d_{i}\;|\;\lambda ,\theta_{r},\delta \right)\pi_{r}\right)}^{Z_{ir}}. \end{array}$$

The expected complete data log likelihood under q(Z)=P(Z|Y,T,d) is

(39) $$\begin{array}{cc} & \sum_{Z}q\left(Z\right)\log L\left(\Theta ,\lambda |Y,T,d,Z\right)\\ = & \sum_{i=1}^{n}\sum_{r=1}^{R}\gamma \left(Z_{ir}\right)\left\{\log\pi_{r}+\log P\left(Y_{i}\;|\;\theta_{r},\alpha \right)+\log P\left(T_{i},d_{i}\;|\;\lambda ,\theta_{r},\delta \right)\right\} \end{array}$$

where $\gamma (Z_{ir})$, $P\left(Y_{i}\;|\;\theta_{r},\alpha \right)$ and $P\left(T_{i},d_{i}\;|\;\lambda ,\theta_{r},\delta \right)$ are defined in equations (42), (34) and (36) respectively.

To estimate all parameters and the baseline hazards simultaneously, we combine the EM algorithm and the method of nonparametric maximum likelihood.

### 3.1. Estimation Procedure: Profile Likelihood with EM Algorithm

**Baseline** **Hazard** **Estimation:** Before starting the EM-step, we profile out the baseline hazard function $\lambda_{0}\left(t\right)$. The survival part of Equation (39) can be separately maximized with respect to λ:

(40) $$\begin{array}{cc} \sum_{i=1}^{n}\sum_{r=1}^{R} & \gamma \left(Z_{ir}\right)\log P\left(T_{i},d_{i}\;|\;\lambda ,\theta_{r},\delta \right)\\ & =\sum_{i=1}^{n}\sum_{r=1}^{R}\gamma \left(Z_{ir}\right)\left\{d_{i}\left(\log\lambda_{i}+\theta_{r}\delta_{0}+X_{i}\delta_{1}\right)-\sum_{p\leq i}\lambda_{p}\exp\left(\theta_{r}\delta_{0}+X_{i}\delta_{1}\right)\right\}. \end{array}$$

By solving $\frac{\partial }{\partial \lambda_{l}}\sum_{i=1}^{n}\sum_{r=1}^{R}\gamma \left(Z_{ir}\right)\log P\left(T_{i},d_{i}\;|\;\lambda ,\theta_{r},\delta \right)=0$, l=1,…,n, we find the maximizer ${\hat{\lambda }}_{l}$ of (40) by holding (θ,δ) fixed, and it is given by

(41) $$\begin{array}{cc} {\hat{\lambda }}_{l}\left(\theta ,\delta \right) & =\frac{d_{i}}{\sum_{p\geq i}\sum_{r=1}^{R}\gamma \left(Z_{pr}\right)\exp\left(\theta_{r}\delta_{0}+X_{p}\delta_{1}\right)}. \end{array}$$

Denote $\hat{\lambda }\left(\theta ,\delta \right)=\left({\hat{\lambda }}_{1}\left(\theta ,\delta \right),\ldots ,{\hat{\lambda }}_{n}\left(\theta ,\delta \right)\right)$.

**The** **E-step:** In the E-step, we use the current parameter estimates Θ=(θ,α,δ) to find the expected values of $Z_{ir}$:

(42) $$\begin{array}{ccc} \gamma \left(Z_{ir}\right)=E\left(Z_{ir}|\;Y_{i},T_{i},d_{i}\right) & = & \frac{\pi_{r}\;P\left(Y_{i}\;|\;\theta_{r},\alpha \right)P\left(T_{i},d_{i}\;|\;\hat{\lambda }\left(\theta ,\delta \right),\theta_{r},\delta \right)}{\sum_{g=1}^{R}\pi_{g}\;P\left(Y_{i}\;|\;\theta_{g},\alpha \right)P\left(T_{i},d_{i}\;|\;\hat{\lambda }\left(\theta ,\delta \right),\theta_{g},\delta \right)}. \end{array}$$

**The** **M-step:** In the M-step, we maximize Equation (39) with respect to $\pi_{r}$ and Θ=(θ,α,δ). Due to the fact that there is no relationship between $\pi_{r}$ and Θ, they can be estimated separately.

1. Calculate the estimates of $\pi_{r}$
  $$\begin{array}{ccc} \hat{\pi_{r}} & = & \frac{\sum_{i=1}^{n}\gamma \left(Z_{ir}\right)}{n}. \end{array}$$
2. We maximize the second and third parts of Equation (39) (with $\hat{\lambda }\left(\theta ,\delta \right)$ in the place of λ)
  (43) $$\begin{array}{c} \sum_{i=1}^{n}\sum_{r=1}^{R}\gamma \left(Z_{ir}\right)\left\{\log P\left(Y_{i}\;|\;\theta_{r},\alpha \right)+\log P\left(T_{i},d_{i}\;|\;\hat{\lambda }\left(\theta ,\delta \right),\theta_{r},\delta \right)\right\} \end{array}$$
  with respect to Θ=(θ,α,δ) to obtain $\hat{\Theta }$.

The estimated parameters from the M-step are returned into the E-step until the value of $\hat{\Theta }$ converges.

### 3.2. Asymptotic Normality of the Profile Likelihood MLE and Its Asymptotic Variance

From (41), an estimator of the cumulative hazard function in the counting process notation is

$$\hat{\Lambda }\left(t\right)=\int_{0}^{t}\frac{\sum_{i=1}^{n}dN_{i}\left(u\right)}{\sum_{i=1}^{n}Y_{i}\left(u\right)\sum_{r=1}^{R}\gamma \left(Z_{ir}\right)\exp\left(\theta_{r}\delta_{0}+X_{i}\delta_{1}\right)}$$

where $N_{i}\left(u\right)=1_{\left\{T_{i}\leq u,d_{i}=1\right\}}$ and $Y_{i}\left(u\right)=1_{\left\{T_{i}\geq u\right\}}$.

Let us denote $E_{F_{n}}f=\int fdF_{n}$. Then the above $\hat{\Lambda }\left(t\right)$ can be written as

(44) $$\begin{array}{c} \hat{\Lambda }\left(t;\Theta ,F_{n}\right)=\int_{0}^{t}\frac{E_{F_{n}}dN\left(u\right)}{E_{F_{n}}Y\left(u\right)\sum_{r=1}^{R}\gamma \left(Z_{r}\right)\exp\left(\theta_{r}\delta_{0}+X\delta_{1}\right)} \end{array}$$

where $N\left(u\right)=1_{\left\{T\leq u,d=1\right\}}$, $Y\left(u\right)=1_{\left\{T\geq u\right\}}$.

Note: From now on we will deal with the cumulative hazard function $\Lambda \left(t\right)=\int_{0}^{t}\lambda \left(s\right)ds$ instead of the hazard function λ(t). The function $\hat{\lambda }\left(\theta ,\delta \right)$ in (42) and (43) will be replaced with $\hat{\Lambda }\left(\Theta ,F_{n}\right)$.

Note: Note that the function $\hat{\Lambda }$ in (44) depends on $\gamma (Z_{r})$. On the other hand, the function $\gamma (Z_{r})$ in (42) depends on $\hat{\Lambda }$. This circular relationship shows the function $\hat{\Lambda }$ in (44) is an implicit function.

Equation (43) gives the profile likelihood function for Θ=(θ,α,δ). The log-profile likelihood function for one observation is

(45) $$\begin{array}{c} \log P\left(Y_{i},T_{i},d_{i}|\Theta ,F_{n}\right)=\sum_{r=1}^{R}\gamma \left(Z_{ir}\right)\left\{\log P\left(Y_{i}\;|\;\theta_{r},\alpha \right)+\log P\left(T_{i},d_{i}\;|\;\hat{\Lambda }\left(\Theta ,F_{n}\right),\theta_{r},\delta \right)\right\} \end{array}$$

where

(46) $$\begin{array}{cc} \sum_{r=1}^{R} & \gamma \left(Z_{ir}\right)\log P\left(Y_{i}\;|\;\theta_{r},\alpha \right)\\ & =\sum_{r=1}^{R}\sum_{m=1}^{M_{i}}\sum_{j=1}^{J}\sum_{\ell =1}^{L}\gamma \left(Z_{ir}\right)Y_{ijm\ell }\left\{a_{\ell }+\phi_{\ell }\left(b_{j}+\theta_{r}\right)-\log\left(1+\sum_{k=2}^{L}\exp\left(a_{k}+\phi_{k}\left(b_{j}+\theta_{r}\right)\right)\right)\right\}, \end{array}$$

and

(47) $$\begin{array}{cc} & \sum_{r=1}^{R}\gamma \left(Z_{ir}\right)\log P\left(T_{i},d_{i}\;|\;\hat{\Lambda }\left(\Theta ,F_{n}\right),\theta_{r},\delta \right)\\ = & \sum_{r=1}^{R}\gamma \left(Z_{r}\right)\left\{d_{i}\left(\log\frac{E_{F_{n}}dN\left(T_{i}\right)}{E_{F_{n}}Y\left(T_{i}\right)\sum_{r^{\prime }=1}^{R}\gamma \left(Z_{r^{\prime }}\right)\exp\left(\theta_{r^{\prime }}\delta_{0}+X\delta_{1}\right)}+\theta_{r}\delta_{0}+X_{i}\delta_{1}\right)\right.\\ & \left.-\exp\left(\theta_{r}\delta_{0}+X_{i}\delta_{1}\right)\int_{0}^{T_{i}}\frac{E_{F_{n}}dN\left(u\right)}{E_{F_{n}}Y\left(u\right)\sum_{r^{\prime }=1}^{R}\gamma \left(Z_{r^{\prime }}\right)\exp\left(\theta_{r^{\prime }}\delta_{0}+X\delta_{1}\right)}\right\}. \end{array}$$

In the above log-likelihood we set $a_{1}=b_{1}=\phi_{1}=\theta_{1}=0$.

**Score** **functions**: The score functions for the profile likelihood are

(48) $$\begin{array}{cc} \phi & \left(Y_{i},T_{i},d_{i}|\Theta ,F_{n}\right)=\phi_{O}\left(Y_{i}|\Theta \right)+\phi_{S}\left(T_{i},d_{i}|\Theta ,F_{n}\right)\\ & =\sum_{r=1}^{R}\gamma \left(Z_{ir}\right)\frac{\partial }{\partial \Theta }\log P\left(Y_{i}\;|\;\theta_{r},\alpha \right)+\sum_{r=1}^{R}\gamma \left(Z_{ir}\right)\frac{\partial }{\partial \Theta }\log P\left(T_{i},d_{i}\;|\;\hat{\Lambda }\left(\Theta ,F_{n}\right),\theta_{r},\delta \right),\\ \psi & \left(Y_{i},T_{i},d_{i}|\Theta ,F_{n}\right)=\sum_{r=1}^{R}\gamma \left(Z_{ir}\right)d_{F}\log P\left(T_{i},d_{i}\;|\;\hat{\Lambda }\left(\Theta ,F_{n}\right),\theta_{r},\delta \right). \end{array}$$

Here all derivatives are calculated treating $\gamma (Z_{ir})$ as constant. We call $\phi_{O}$ is the score function for the ordinal response model and $\phi_{S}$ is the one for the survival model.

**Theorem** **3.** *(The efficient score function) We drop subscript i in Equation (48). We have the followings: at the true value of (Θ,F),*

*1.*  *$\hat{\Lambda }\left(t;\Theta ,F\right)=\Lambda \left(t\right)$, the true cumulative hazard function, and;*
*2.*  *the score function ϕ(Y,T,d|Θ,F) defined in (48) is the efficient score function in the model.*
  The proof is given in Appendix A.

**Theorem** **4.** *(Asymptotic normality of the profile likelihood MLE) We assume the followings:*

(*A1*) *The maximal right-censoring time τ>0 is finite and satisfies S(τ)=P(T>τ)>0.*
(*A2*) *The covariate X is bounded and the parameter Θ=(θ,α,δ) is in a compact set. This implies that, for some 0<M<∞, we have ∥X∥≤M and ∥Θ∥≤M.*
(*A3*) *The empirical cdf $F_{n}$ is $\sqrt{n}$ consistent: $\sqrt{n}\left(F_{n}-F_{0}\right)=O_{p}\left(1\right)$.*
(*A4*) *The efficient information matrix $\tilde{I}$ is invertible.*

*Then the profile likelihood MLE ${\hat{\Theta }}_{n}$ obtained by the procedure described in Section 3.1 is asymptotically normal:*

$$\sqrt{n}\left({\hat{\Theta }}_{n}-\Theta_{0}\right)∼N\left(0,{\tilde{I}}^{-1}\right),$$

*where $\tilde{I}=E\left(\phi \phi^{T}\right)$ is the efficient information with ϕ is defined in (48).*

**Proof.**  We check conditions (R1)-(R4) in Section 2.4 so that Theorem 1 and Theorem 2 can be used to get result stated in the theorem.

Since the ordinal response data part is a parametric model, we mainly discuss for the survival part of the model. The survival part of the profile log -likelihood function for a one observation is given in (47).

To express the survival part of the score function $\phi_{S}\left(T,d|\Theta ,F\right)$ in the form given in condition (R4), we introduce a few notations.

Let

(49) $$\begin{array}{ccc} \gamma (Z_{r}|\Theta ,\Lambda ) & = & \frac{\pi_{r}\;P\left(Y\;|\;\theta_{r},\alpha \right)P\left(T,d\;|\;\Lambda ,\theta_{r},\delta \right)}{\sum_{g=1}^{R}\pi_{g}\;P\left(Y\;|\;\theta_{g},\alpha \right)P\left(T,d\;|\;\Lambda ,\theta_{g},\delta \right)}. \end{array}$$

The function $\gamma (Z_{r}|\Theta ,\Lambda )$ is differentiable with respect to Θ and Λ. Then the function $\gamma (Z_{r})$ in (42) can be expressed as

$$\gamma \left(Z_{r}\right)=\gamma \left(Z_{r}|\Theta ,\hat{\Lambda }\left(\Theta ,F\right)\right).$$

Let

$$\begin{array}{ccc} M_{0}\left(t|\Theta ,F,\Lambda \right) & = & E_{F}Y\left(u\right)\sum_{r=1}^{R}\gamma \left(Z_{r}|\Theta ,\Lambda \right)\exp\left(\theta_{r}\delta_{0}+X\delta_{1}\right)\\ M_{1}\left(t|\Theta ,F,\Lambda \right) & = & E_{F}Y\left(u\right)\sum_{r=1}^{R}\gamma \left(Z_{r}|\Theta ,\Lambda \right)\left(\begin{array}{c} \delta_{0}\\ \theta_{r}\\ X \end{array}\right)\exp\left(\theta_{r}\delta_{0}+X\delta_{1}\right). \end{array}$$

Then the score function for the survival part $\phi_{S}\left(T,d|\Theta ,F\right)$ is

(50) $$\begin{array}{cc} & {\tilde{\phi }}_{S}\left(T,d|\Theta ,F,\hat{\Lambda }\left(\Theta ,F\right)\right)\\ = & \sum_{r=1}^{R}\gamma \left(Z_{r}|\Theta ,\hat{\Lambda }\left(\Theta ,F\right)\right)\frac{\partial }{\partial \Theta }\log P\left(T,d\;|\;\hat{\Lambda }\left(\Theta ,F\right),\theta_{r},\delta \right)\\ = & \sum_{r=1}^{R}\gamma \left(Z_{r}|\Theta ,\hat{\Lambda }\left(\Theta ,F\right)\right)\left\{d\left[\left(\begin{array}{c} \delta_{0}\\ \theta_{r}\\ X \end{array}\right)-\frac{M_{1}\left(T|\Theta ,F,\hat{\Lambda }\left(\Theta ,F\right)\right)}{M_{0}\left(T|\Theta ,F,\hat{\Lambda }\left(\Theta ,F\right)\right)}\right]\right.\\ & \left.+\exp\left(\theta_{r}\delta_{0}+X\delta_{1}\right)\int_{0}^{T}\frac{E_{F}dN\left(u\right)}{M_{0}\left(u|\Theta ,F,\hat{\Lambda }\left(\Theta ,F\right)\right)}\left[\left(\begin{array}{c} \delta_{0}\\ \theta_{r}\\ X \end{array}\right)-\frac{M_{1}\left(u|\Theta ,F,\hat{\Lambda }\left(\Theta ,F\right)\right)}{M_{0}\left(u|\Theta ,F,\hat{\Lambda }\left(\Theta ,F\right)\right)}\right]\right\}. \end{array}$$

We will check condition (R4) using the function defined by

(51) $$\begin{array}{cc} & {\tilde{\phi }}_{S}\left(T,d|\Theta ,F,\Lambda \right)\\ = & \sum_{r=1}^{R}\gamma \left(Z_{r}|\Theta ,\Lambda \right)\left\{d\left[\left(\begin{array}{c} \delta_{0}\\ \theta_{r}\\ X \end{array}\right)-\frac{M_{1}\left(T|\Theta ,F,\Lambda \right)}{M_{0}\left(T|\Theta ,F,\Lambda \right)}\right]\right.\\ & \left.+\exp\left(\theta_{r}\delta_{0}+X\delta_{1}\right)\int_{0}^{T}\frac{E_{F}dN\left(u\right)}{M_{0}\left(u|\Theta ,F,\Lambda \right)}\left[\left(\begin{array}{c} \delta_{0}\\ \theta_{r}\\ X \end{array}\right)-\frac{M_{1}\left(u|\Theta ,F,\Lambda \right)}{M_{0}\left(u|\Theta ,F,\Lambda \right)}\right]\right\}. \end{array}$$

 □

**Condition (R1):** With assumptions (A1) and (A2), a straight forward calculation can show that the probability functions $P(T,d|\lambda ,\theta_{r},\delta )$ in (36) and $P(Y|\theta_{r},\alpha )$ in (34) are bounded from below.

We calculated the survival part score function $\phi_{S}\left(T,d|\Theta ,F\right)={\tilde{\phi }}_{S}\left(T,d|\Theta ,F,\hat{\Lambda }\left(\Theta ,F\right)\right)$ in (50). The ordinal response data part is a parametric model, it is differentiable with respect to the parameter Θ (we omit the calculation).

We calculate the score function $\psi \left(T,d|\Theta ,F\right)=\sum_{r=1}^{R}\gamma \left(Z_{ir}\right)d_{F}\log P\left(T,d\;|\;\hat{\Lambda }\left(\Theta ,F\right),\theta_{r},\delta \right)$. For an integrable function *h* with the same domain as the cdfs *F*,

$$\begin{array}{ccc} \psi (Y,T,d|\Theta ,F)h & = & \sum_{r=1}^{R}\gamma \left(Z_{r}\right)d_{F}\log P\left(T,d\;|\;\hat{\Lambda }\left(\Theta ,F\right),\theta_{r},\delta \right)h\\ & = & \sum_{r=1}^{R}\gamma \left(Z_{r}\right)\left\{d\left(\frac{E_{h}dN\left(T\right)}{E_{F}dN\left(T\right)}-\frac{E_{h}Y\left(T\right)\sum_{r=1}^{R}\gamma \left(Z_{r}\right)\exp\left(\theta_{r}\delta_{0}+X\delta_{1}\right)}{E_{F}Y\left(T\right)\sum_{r=1}^{R}\gamma \left(Z_{r}\right)\exp\left(\theta_{r}\delta_{0}+X\delta_{1}\right)}\right)\right.\\ & & -\exp\left(\theta_{r}\delta_{0}+X\delta_{1}\right)\int_{0}^{T}\frac{E_{h}dN\left(u\right)}{E_{F}Y\left(u\right)\sum_{r=1}^{R}\gamma \left(Z_{r}\right)\exp\left(\theta_{r}\delta_{0}+X\delta_{1}\right)}\\ & & \left.+\exp\left(\theta_{r}\delta_{0}+X\delta_{1}\right)\int_{0}^{T}\frac{E_{F}dN\left(u\right)E_{h}Y\left(u\right)\sum_{r=1}^{R}\gamma \left(Z_{r}\right)\exp\left(\theta_{r}\delta_{0}+X\delta_{1}\right)}{{\left(E_{F}Y\left(u\right)\sum_{r=1}^{R}\gamma \left(Z_{r}\right)\exp\left(\theta_{r}\delta_{0}+X\delta_{1}\right)\right)}^{2}}\right\}. \end{array}$$

**Condition (R2):** In (A3), we assumed $\sqrt{n}\left(F_{n}-F_{0}\right)=O_{p}\left(1\right)$ ($\sqrt{n}$-consistency of $F_{n}$). In Appendix C, we showed consistencies of ${\hat{\Theta }}_{n}$ and $\hat{\Lambda }\left({\hat{\Theta }}_{n},F_{n}\right)$: ${\hat{\Theta }}_{n}-\Theta_{0}=o_{p}\left(1\right)$ and $\hat{\Lambda }\left({\hat{\Theta }}_{n},F_{n}\right)-\Lambda_{0}=o_{p}\left(1\right)$. In Theorem 3 we verified the rest of conditions in (R2).

**Condition (R3):** In (A4), we assumed the efficient information matrix $\tilde{I}$ is invertible.

**Condition (R4):** The score function ${\tilde{\phi }}_{S}\left(T,d|\Theta ,F,\Lambda \right)$ given in (51) is differentiable with respect to the parameters (Θ,F,Λ) (with assumptions (A1) and (A2), the derivative is a bounded function). It follows Condition (R4).

## 4. Conclusions

We proposed a “statistical generalized derivative” to deal with an implicit function that appears in the profile likelihood estimation in semiparametric model examples. The example includes the joint model of ordinal responses and the proportional hazards model with a finite mixture which we treated in this paper. Using the “statistical generalized derivative” we expanded the profile likelihood function and showed asymptotic normality of the profile likelihood MLE. Moreover, this approach enabled us to express the SE of the estimator in terms of the profile likelihood score function. This contributes to the profile likelihood estimation methods where, otherwise, no direct expansions of the profile likelihood were possible. Our approach can be applied to not only the example in this paper, but also to many models with implicit function in the profile likelihood estimation. As a next step, we are working on applying our method to those examples.

## Appendix A. Proof of Theorem 3 (The Efficient Score Function)

**Proof.** For ease notation we denote the true values of the parameters by (Θ,F,Λ). From (44), replacing $F_{n}$ by *F*, we have

$$\hat{\Lambda }\left(t;\Theta ,F\right)=\int_{0}^{t}\frac{EdN(u)}{EY\left(u\right)\sum_{r=1}^{R}\gamma \left(Z_{r}\right)\exp\left(\theta_{r}\delta_{0}+X\delta_{1}\right)}$$

where *E* is the expectation with respect to the true distribution *F*. Since, at the true value of the parameters (Θ,F,Λ),

(A1) $$\begin{array}{ccc} EdN(u) & = & E\left[Y\left(u\right)\sum_{r=1}^{R}\gamma \left(Z_{r}\right)\exp\left(\theta_{r}\delta_{0}+X\delta_{1}\right)\right]d\Lambda \left(u\right), \end{array}$$

we have that $\hat{\Lambda }\left(t;\Theta ,F\right)=\Lambda \left(t\right)$. The score function $\phi \left(Y,T,d|\Theta ,F\right)=\phi_{O}\left(Y|\Theta \right)+\phi_{S}\left(T,d|\Theta ,F\right)$ in (48) has two parts: the score function for the ordinal response model $\phi_{O}\left(Y|\Theta \right)$ and the score function for the survival model $\phi_{S}\left(T,d|\Theta ,F\right)$. Since the score function for the ordinal response model does not involve the parameter Λ, we will only work on the survival part of score function. We treat the part $\gamma (Z_{r})$ as constant in terms of the parameters. Let

(A2) $$\begin{array}{ccc} M_{1}\left(t\right) & = & E\sum_{r=1}^{R}\gamma \left(Z_{r}\right)\left(\begin{array}{c} \delta_{0}\\ \theta_{r}\\ X \end{array}\right)\exp\left(\theta_{r}\delta_{0}+X\delta_{1}\right)I\left(t\leq T\right)\\ M_{0}\left(t\right) & = & E\sum_{r=1}^{R}\gamma \left(Z_{r}\right)\exp\left(\theta_{r}\delta_{0}+X\delta_{1}\right)I\left(t\leq T\right) \end{array}$$

Then the score function in the survival part of the model at the true value of parameters Θ and *F* is

(A3) $$\begin{array}{cc} & \phi_{S}\left(T,d|\Theta ,F\right)=\sum_{r=1}^{R}\gamma \left(Z_{r}\right)\frac{\partial }{\partial \Theta }\log P\left(T,d\;|\;\hat{\Lambda }\left(\Theta ,F\right),\theta_{r},\delta \right)\\ = & \sum_{r=1}^{R}\gamma \left(Z_{r}\right)\frac{\partial }{\partial \Theta }\left\{d\left(\log\frac{EdN(T)}{EY\left(T\right)\sum_{r=1}^{R}\gamma \left(Z_{r}\right)\exp\left(\theta_{r}\delta_{0}+X\delta_{1}\right)}+\theta_{r}\delta_{0}+X\delta_{1}\right)\right.\\ & \left.-\exp\left(\theta_{r}\delta_{0}+X\delta_{1}\right)\int_{0}^{T}\frac{EdN(u)}{EY\left(u\right)\sum_{r=1}^{R}\gamma \left(Z_{r}\right)\exp\left(\theta_{r}\delta_{0}+X\delta_{1}\right)}\right\}\\ = & \sum_{r=1}^{R}\gamma \left(Z_{r}\right)d\left[\left(\begin{array}{c} \delta_{0}\\ \theta_{r}\\ X \end{array}\right)-\frac{M_{1}\left(T\right)}{M_{0}\left(T\right)}\right]\\ & -\sum_{r=1}^{R}\gamma \left(Z_{r}\right)\left(\exp\left(\theta_{r}\delta_{0}+X\delta_{1}\right)\int_{0}^{T}\left[\left(\begin{array}{c} \delta_{0}\\ \theta_{r}\\ X \end{array}\right)-\frac{M_{1}\left(u\right)}{M_{0}\left(u\right)}\right]d\Lambda \left(u\right)\right) \end{array}$$

where we used Equation (A1). The last expression is the efficient score function in the survival part of the model derived in Equation (A4), Appendix B.  □

## Appendix B. Derivation of Efficient Score Function in the Joint Model

In this appendix, we derive the efficient score function in the joint model using (8). We denote $P_{r,\Theta ,\Lambda }\left(T,d\right)=P\left(T,d\;|\;\Lambda ,\theta_{r},\delta \right)$. Again the true values of the parameters are denoted by (Θ,F,Λ).

The survival part of log-likelihood function for a one observation is

$$\begin{array}{c} \sum_{r=1}^{R}\gamma \left(Z_{r}\right)\log P_{r,\Theta ,\Lambda }\left(T,d\right)=\sum_{r=1}^{R}\gamma \left(Z_{r}\right)\left\{d\left(\log\lambda \left(T\right)+\theta_{r}\delta_{0}+X\delta_{1}\right)-\Lambda \left(T\right)\exp\left(\theta_{r}\delta_{0}+X\delta_{1}\right)\right\}. \end{array}$$

The score function for Θ is

$$\begin{array}{ccc} {\dot{\ell }}_{\Theta ,\Lambda } & = & \sum_{r=1}^{R}\gamma \left(Z_{r}\right)\frac{\partial }{\partial \Theta }\log P_{r,\Theta ,\Lambda }\left(T,d\right)=\sum_{r=1}^{R}\gamma \left(Z_{r}\right)\left(\begin{array}{c} \delta_{0}\\ \theta_{r}\\ X \end{array}\right)\left\{d-\Lambda \left(T\right)\exp\left(\theta_{r}\delta_{0}+X\delta_{1}\right)\right\}. \end{array}$$

Let h:[0,τ]→R be a function on [0,τ]. The path defined by

$$d\Lambda_{s}=\left(1+sh\right)d\Lambda$$

is a submodel passing through Λ at s=0. The corresponding path for the λ is

$$\lambda_{s}\left(t\right)=\frac{d\Lambda_{s}\left(t\right)}{dt}=\left(1+sh\right)\lambda \left(t\right).$$

The derivative of the log-likelihood function

$$\begin{array}{c} \sum_{r=1}^{R}\gamma \left(Z_{r}\right)\log P_{r,\Theta ,\Lambda_{s}}\left(T,d\right)=\sum_{r=1}^{R}\gamma \left(Z_{r}\right)\left\{d\left(\log\lambda_{s}\left(T\right)+\theta_{r}\delta_{0}+X\delta_{1}\right)-\Lambda_{s}\left(T\right)\exp\left(\theta_{r}\delta_{0}+X\delta_{1}\right)\right\}. \end{array}$$

with respect to *s* at s=0 is the score operator for Λ:

$$B_{\Theta ,\Lambda }h=\frac{d}{ds}{|}_{s=0}\sum_{r=1}^{R}\gamma \left(Z_{r}\right)\log P_{r,\Theta ,\Lambda_{s}}\left(T,d\right)=\sum_{r=1}^{R}\gamma \left(Z_{r}\right)\left(dh\left(T\right)-\exp\left(\theta_{r}\delta_{0}+X\delta_{1}\right)\int_{0}^{T}h\left(u\right)d\Lambda \left(u\right)\right).$$

**Information operator $B_{\Theta ,\Lambda }^{*}B_{\Theta ,\Lambda }$**

For functions g,h:[0,τ]→R, define a paths $d\Lambda_{s,t}=\left(1+sg+th+stgh\right)d\Lambda$. Then

$$\begin{array}{c} \frac{\partial }{\partial s}{|}_{\left(s,t)=(0,0\right)}\sum_{r=1}^{R}\gamma \left(Z_{r}\right)\log P_{r,\Theta ,\Lambda_{s,t}}\left(T,d\right)=B_{\Theta ,\Lambda }g,\\ \frac{\partial }{\partial t}{|}_{\left(s,t)=(0,0\right)}\sum_{r=1}^{R}\gamma \left(Z_{r}\right)\log P_{r,\Theta ,\Lambda_{s,t}}\left(T,d\right)=B_{\Theta ,\Lambda }h. \end{array}$$

Using these we have

$$\begin{array}{ccc} {\langle B_{\Theta ,\Lambda }g,B_{\Theta ,\Lambda }h\rangle }_{L_{2}\left(P\right)} & = & E\{\left(B_{\Theta ,\Lambda }g\right)\left(B_{\Theta ,\Lambda }h\right)\}\\ & = & -E\left\{\frac{\partial^{2}}{\partial t\partial s}{|}_{\left(s,t)=(0,0\right)}\sum_{r=1}^{R}\gamma \left(Z_{r}\right)\log P_{r,\Theta ,\Lambda_{s,t}}\left(T,d\right)\right\}\\ & = & -E\left\{\frac{\partial }{\partial t}{|}_{t=0}B_{\Theta ,\Lambda_{0,t}}g\right\}\\ & = & E\left\{\sum_{r=1}^{R}\gamma \left(Z_{r}\right)\exp\left(\theta_{r}\delta_{0}+X\delta_{1}\right)\int_{0}^{\tau }I\left(u\leq T\right)g\left(u\right)h\left(u\right)d\Lambda \left(u\right)\right\}\\ & = & \int_{0}^{\tau }h\left(u\right)E\sum_{r=1}^{R}\gamma \left(Z_{r}\right)\exp\left(\theta_{r}\delta_{0}+X\delta_{1}\right)I\left(u\leq T\right)g\left(u\right)d\Lambda \left(u\right)\\ & = & {\langle E\sum_{r=1}^{R}\gamma \left(Z_{r}\right)\exp\left(\theta_{r}\delta_{0}+X\delta_{1}\right)I\left(u\leq T\right)g\left(u\right),\;h\left(u\right)\rangle }_{L_{2}\left(\Lambda \right)} \end{array}$$

Since

$${\langle B_{\Theta ,\Lambda }g,B_{\Theta ,\Lambda }h\rangle }_{L_{2}\left(P\right)}={\langle B_{\Theta ,\Lambda }^{*}B_{\Theta ,\Lambda }g,h\rangle }_{L_{2}\left(\Lambda \right)},$$

we have the information operator

$$B_{\Theta ,\Lambda }^{*}B_{\Theta ,\Lambda }g=E\sum_{r=1}^{R}\gamma \left(Z_{r}\right)\exp\left(\theta_{r}\delta_{0}+X\delta_{1}\right)I\left(t\leq T\right)g\left(t\right).$$

Since the operator multiplies a number, the inverse is

$${\left(B_{\Theta ,\Lambda }^{*}B_{\Theta ,\Lambda }\right)}^{-1}g={\left[E\sum_{r=1}^{R}\gamma \left(Z_{r}\right)\exp\left(\theta_{r}\delta_{0}+X\delta_{1}\right)I\left(t\leq T\right)\right]}^{-1}g\left(t\right).$$

**Calculation of $B_{\Theta ,\Lambda }^{*}{\dot{\ell }}_{\Theta ,\Lambda }$**

Consider a paths $\left(s,t\right)\to \left(\Theta +sa,\Lambda_{t}\right)$ with $d\Lambda_{t}=\left(1+th\right)d\Lambda$. Then

$$\begin{array}{ccc} \frac{\partial }{\partial s}{|}_{\left(s,t)=(0,0\right)}\sum_{r=1}^{R}\gamma \left(Z_{r}\right)\log P_{r,\Theta +sa,\Lambda_{t}}\left(T,d\right) & = & a^{T}{\dot{\ell }}_{\Theta ,\Lambda }\\ \frac{\partial }{\partial t}{|}_{\left(s,t)=(0,0\right)}\sum_{r=1}^{R}\gamma \left(Z_{r}\right)\log P_{r,\Theta +sa,\Lambda_{t}}\left(T,d\right) & = & B_{\Theta ,\Lambda }h. \end{array}$$

Using these we compute that

$$\begin{array}{ccc} {\langle a^{T}{\dot{\ell }}_{\Theta ,\Lambda },B_{\Theta ,\Lambda }h\rangle }_{L_{2}\left(P\right)} & = & E\{\left(a^{T}{\dot{\ell }}_{\Theta ,\Lambda }\right)\left(B_{\Theta ,\Lambda }h\right)\}\\ & = & -E\left\{\frac{\partial^{2}}{\partial t\partial s}{|}_{\left(s,t)=(0,0\right)}\sum_{r=1}^{R}\gamma \left(Z_{r}\right)\log P_{r,\Theta +sa,\Lambda_{t}}\left(T,d\right)\right\}\\ & = & -E\left\{\frac{\partial }{\partial t}{|}_{t=0}a^{T}{\dot{\ell }}_{\Theta ,\Lambda_{t}}\right\}\\ & = & a^{T}E\sum_{r=1}^{R}\gamma \left(Z_{r}\right)\left(\begin{array}{c} \delta_{0}\\ \theta_{r}\\ X \end{array}\right)\left\{\exp\left(\theta_{r}\delta_{0}+X\delta_{1}\right)\int_{0}^{\tau }I\left(u\leq T\right)h\left(u\right)d\Lambda \left(u\right)\right\}\\ & = & a^{T}\int_{0}^{\tau }E\sum_{r=1}^{R}\gamma \left(Z_{r}\right)\left(\begin{array}{c} \delta_{0}\\ \theta_{r}\\ X \end{array}\right)\exp\left(\theta_{r}\delta_{0}+X\delta_{1}\right)I\left(u\leq T\right)h\left(u\right)d\Lambda \left(u\right)\\ & = & {\langle a^{T}E\sum_{r=1}^{R}\gamma \left(Z_{r}\right)\left(\begin{array}{c} \delta_{0}\\ \theta_{r}\\ X \end{array}\right)\exp\left(\theta_{r}\delta_{0}+X\delta_{1}\right)I\left(u\leq T\right),\;h\rangle }_{L_{2}\left(\Lambda \right)} \end{array}$$

Since

$${\langle a^{T}{\dot{\ell }}_{\Theta ,\Lambda },B_{\Theta ,\Lambda }h\rangle }_{L_{2}\left(P\right)}={\langle a^{T}B_{\Theta ,\Lambda }^{*}{\dot{\ell }}_{\Theta ,\Lambda },h\rangle }_{L_{2}\left(\Lambda \right)},$$

we have that

$$B_{\Theta ,\Lambda }^{*}{\dot{\ell }}_{\Theta ,\Lambda }=E\sum_{r=1}^{R}\gamma \left(Z_{r}\right)\left(\begin{array}{c} \delta_{0}\\ \theta_{r}\\ X \end{array}\right)\exp\left(\theta_{r}\delta_{0}+X\delta_{1}\right)I\left(u\leq T\right).$$

**Efficient score function**

Then the efficient score function for the survival part of the model is given by

(A4) $$\begin{array}{ccc} {\tilde{\ell }}_{\Theta ,\Lambda } & = & {\dot{\ell }}_{\Theta ,\Lambda }-B_{\Theta ,\Lambda }{\left(B_{\Theta ,\Lambda }^{*}B_{\Theta ,\Lambda }\right)}^{-1}B_{\Theta ,\Lambda }^{*}{\dot{\ell }}_{\Theta ,\Lambda }\\ & = & \sum_{r=1}^{R}\gamma \left(Z_{r}\right)\left(\begin{array}{c} \delta_{0}\\ \theta_{r}\\ X \end{array}\right)\left\{d-\Lambda \left(T\right)\exp\left(\theta_{r}\delta_{0}+X\delta_{1}\right)\right\}-B_{\Theta ,\Lambda }\frac{M_{1}\left(t\right)}{M_{0}\left(t\right)}\\ & = & \sum_{r=1}^{R}\gamma \left(Z_{r}\right)\left(\begin{array}{c} \delta_{0}\\ \theta_{r}\\ X \end{array}\right)\left\{d-\Lambda \left(T\right)\exp\left(\theta_{r}\delta_{0}+X\delta_{1}\right)\right\}\\ & & -\sum_{r=1}^{R}\gamma \left(Z_{r}\right)\left(d\frac{M_{1}\left(T\right)}{M_{0}\left(T\right)}-\exp\left(\theta_{r}\delta_{0}+X\delta_{1}\right)\int_{0}^{T}\frac{M_{1}\left(u\right)}{M_{0}\left(u\right)}d\Lambda \left(u\right)\right)\\ & = & \sum_{r=1}^{R}\gamma \left(Z_{r}\right)d\left[\left(\begin{array}{c} \delta_{0}\\ \theta_{r}\\ X \end{array}\right)-\frac{M_{1}\left(T\right)}{M_{0}\left(T\right)}\right]\\ & & -\sum_{r=1}^{R}\gamma \left(Z_{r}\right)\left(\exp\left(\theta_{r}\delta_{0}+X\delta_{1}\right)\int_{0}^{T}\left[\left(\begin{array}{c} \delta_{0}\\ \theta_{r}\\ X \end{array}\right)-\frac{M_{1}\left(u\right)}{M_{0}\left(u\right)}\right]d\Lambda \left(u\right)\right) \end{array}$$

where $M_{1}\left(t\right)$ and $M_{0}\left(t\right)$ are defined in (A2).

## Appendix C. Proof of Consistency

We outline proof of consistencies of ${\hat{\Theta }}_{n}$ and $\hat{\Lambda }\left({\hat{\Theta }}_{n},F_{n}\right)$. These consistencies were required in the verification of Condition (R2) in Theorem 4.

We use the proof of Theorem 5.7 in the book [14] (page 45) and adopt the notations from the book.

**Proof.** Lets us denote the log-likelihood function $M_{n}\left(\Theta ,\Lambda \right)=P_{n}\log P_{\Theta ,\Lambda }$ and the corresponding limit function $M\left(\Theta ,\Lambda \right)=P_{\Theta_{0},\Lambda_{0}}\log P_{\Theta ,\Lambda }$. We denote the MLE and the true value of (Θ,Λ) by $\left({\hat{\Theta }}_{n},{\hat{\Lambda }}_{n}\right)$ and $\left(\Theta_{0},\Lambda_{0}\right)$, respectively. Then by the construction of profile likelihood function

$$M_{n}\left({\hat{\Theta }}_{n},{\hat{\Lambda }}_{n}\right)=M_{n}\left({\hat{\Theta }}_{n},\hat{\Lambda }\left({\hat{\Theta }}_{n},F_{n}\right)\right)$$

and ${\hat{\Lambda }}_{n}=\hat{\Lambda }\left({\hat{\Theta }}_{n},F_{n}\right)$. It follows that the consistency of the MLE $\left({\hat{\Theta }}_{n},{\hat{\Lambda }}_{n}\right)$ implies the consistency of $\left({\hat{\Theta }}_{n},\hat{\Lambda }\left({\hat{\Theta }}_{n},F_{n}\right)\right)$. BY (A2), Θ is bounded and Λ(t) is in the set of monotone increasing function. The parameter (Θ,Λ) is in a set of Donsker class. Since the function $\log P_{\Theta ,\Lambda }$ is differentiable in the parameter (Θ,Λ) with square integrable envelope function, the set $\left\{\log P_{\Theta ,\Lambda }:\left(\Theta ,\Lambda \right)\mathrm{in}\mathrm{the}\mathrm{Donsker}\mathrm{class}\right\}$ is Donsker as well (by Corollary 2.10.13 in [16]). It follows that

$$M_{n}\left({\hat{\Theta }}_{n},{\hat{\Lambda }}_{n}\right)-M\left({\hat{\Theta }}_{n},{\hat{\Lambda }}_{n}\right)=o_{p}\left(1\right).$$

By the property of Kullback-Leibler divergence, we have

$$M\left(\Theta ,\Lambda \right)<M\left(\Theta_{0},\Lambda_{0}\right)$$

for all $\left(\Theta ,\Lambda \right)\neq \left(\Theta_{0},\Lambda_{0}\right)$. By definition of the MLE also we have

$$M_{n}\left({\hat{\Theta }}_{n},{\hat{\Lambda }}_{n}\right)\geq M_{n}\left(\Theta_{0},\Lambda_{0}\right).$$

By the proof of Theorem 5.7 in [14] (page 45), the consistency of the MLE $\left({\hat{\Theta }}_{n},{\hat{\Lambda }}_{n}\right)-\left(\Theta_{0},\Lambda_{0}\right)=o_{p}\left(1\right)$ follows.  □
